# Serial markers of coagulation and inflammation and the occurrence of clinical pulmonary thromboembolism in mechanically ventilated patients with SARS-CoV-2 infection; the prospective Maastricht intensive care COVID cohort

**DOI:** 10.1186/s12959-021-00286-7

**Published:** 2021-05-31

**Authors:** Mark M. G. Mulder, LIoyd Brandts, Renée A. G. Brüggemann, Marcel Koelmann, Alexander S. Streng, Renske H. Olie, Hester A. Gietema, Henri M. H. Spronk, Iwan C. C. van der Horst, Jan-Willem E. M. Sels, Joachim E. Wildberger, Sander M. J. van Kuijk, Ronny M. Schnabel, Hugo ten Cate, Yvonne M. C. Henskens, Bas C. T. van Bussel

**Affiliations:** 1 Department of Intensive Care Medicine, Maastricht University Medical Centre+, Maastricht, The Netherlands; 2 Department of Clinical Epidemiology and Medical Technology Assessment, Maastricht University Medical Centre+, Maastricht, The Netherlands; 3 Department of Internal Medicine, Maastricht University Medical Centre+, Maastricht, The Netherlands; 4 Department of Clinical Chemistry, Central Diagnostic Laboratory, Maastricht University Medical Centre+, Maastricht, The Netherlands; 5 Thrombosis Expert Centre Maastricht and Department of Internal Medicine, Section Vascular Medicine, Maastricht University Medical Centre+, Maastricht, The Netherlands; 6 Cardiovascular Research Institute Maastricht (CARIM), Maastricht University, Maastricht, The Netherlands; 7 Department of Radiology and Nuclear Medicine, Maastricht University Medical Centre+, Maastricht, The Netherlands; 8 GROW School of Oncology and Developmental Biology, Maastricht University, Maastricht, The Netherlands; 9 Department of Cardiology, Maastricht University Medical Centre+, Maastricht, The Netherlands; 10 Care and Public Health Research Institute, Maastricht University Medical Centre+, Maastricht, The Netherlands

**Keywords:** SARS-CoV-2, COVID-19, Pulmonary embolism, Pulmonary thrombosis, Coagulation, D-dimer, Fibrinogen, C-reactive protein, Intensive care

## Abstract

**Background:**

The incidence of pulmonary thromboembolism is high in SARS-CoV-2 patients admitted to the Intensive Care. Elevated biomarkers of coagulation (fibrinogen and D-dimer) and inflammation (c-reactive protein (CRP) and ferritin) are associated with poor outcome in SARS-CoV-2. Whether the time-course of fibrinogen, D-dimer, CRP and ferritin is associated with the occurrence of pulmonary thromboembolism in SARS-CoV-2 patients is unknown. We hypothesise that patients on mechanical ventilation with SARS-CoV-2 infection and clinical pulmonary thromboembolism have lower concentrations of fibrinogen and higher D-dimer, CRP, and ferritin concentrations over time compared to patients without a clinical pulmonary thromboembolism.

**Methods:**

In a prospective study, fibrinogen, D-dimer, CRP and ferritin were measured daily. Clinical suspected pulmonary thromboembolism was either confirmed or excluded based on computed tomography pulmonary angiography (CTPA) or by transthoracic ultrasound (TTU) (i.e., right-sided cardiac thrombus). In addition, patients who received therapy with recombinant tissue plasminogen activator were included when clinical instability in suspected pulmonary thromboembolism did not allow CTPA. Serial data were analysed using a mixed-effects linear regression model, and models were adjusted for known risk factors (age, sex, APACHE-II score, body mass index), biomarkers of coagulation and inflammation, and anticoagulants.

**Results:**

Thirty-one patients were considered to suffer from pulmonary thromboembolism ((positive CTPA (*n* = 27), TTU positive (*n* = 1), therapy with recombinant tissue plasminogen activator (*n* = 3)), and eight patients with negative CTPA were included. After adjustment for known risk factors and anticoagulants, patients with, compared to those without, clinical pulmonary thromboembolism had lower average fibrinogen concentration of − 0.9 g/L (95% CI: − 1.6 – − 0.1) and lower average ferritin concentration of − 1045 μg/L (95% CI: − 1983 – − 106) over time. D-dimer and CRP average concentration did not significantly differ, 561 μg/L (− 6212–7334) and 27 mg/L (− 32–86) respectively. Ferritin lost statistical significance, both in sensitivity analysis and after adjustment for fibrinogen and D-dimer.

**Conclusion:**

Lower average concentrations of fibrinogen over time were associated with the presence of clinical pulmonary thromboembolism in patients at the Intensive Care, whereas D-dimer, CRP and ferritin were not. Lower concentrations over time may indicate the consumption of fibrinogen related to thrombus formation in the pulmonary vessels.

**Supplementary Information:**

The online version contains supplementary material available at 10.1186/s12959-021-00286-7.

## Background

SARS-CoV-2 is highly heterogeneous in its presentation, and a high incidence of pulmonary thromboembolism during mechanical ventilation at the Intensive Care has been reported [1–5]. A SARS-CoV-2 specific trait interacting with host inflammation might be related to the development of pulmonary thromboembolism [6–9]. Biomarkers of coagulation, such as fibrinogen and D-dimer, and biomarkers of inflammation, such as c-reactive protein (CRP) and ferritin, appear higher than reference values SARS-CoV-2 [10–15]. Higher concentrations of single biomarkers have been used to estimate the risk of pulmonary thromboembolism [13, 16–20]. On the other hand, lower fibrinogen concentrations have also been found in non-SARS-CoV-2 patients with acute pulmonary thromboembolism [21–23]. Both lower fibrinogen and higher D-dimer concentrations were associated with a greater load of clot burden [24]. These alterations in coagulation biomarkers reflect activation of coagulation, resulting in fibrinogen consumption in the pulmonary vasculature and the fibrinolytic system driving higher D-dimer concentrations. Moreover, high ferritin concentrations contribute to cytokine release in severe SARS-CoV-2 infection, promoting a hypercoagulable state [25, 26]. However, the causal role for coagulation and inflammation in the disease course is still to be established in SARS-CoV-2 infection, and the association with the occurrence of pulmonary thromboembolism over time is unknown [7, 27, 28]. Serial measurements of these biomarkers over time are required to define the role of biomarkers of coagulation and inflammation as a sign of the risk of pulmonary thromboembolism. Our study aims to unravel the association of serial measurements of fibrinogen, D-dimer, CRP and ferritin with clinical pulmonary thromboembolism in SARS-CoV-2 during Intensive Care stay.

## Materials and methods

### Participants

We initiated the Maastricht Intensive Care COVID (*MaastrICCht*) cohort to prospectively study SARS-CoV-2 patients admitted to the Intensive Care of the Maastricht University Medical Center+ (Maastricht UMC+), a tertiary care university hospital in the southern part of the Netherlands. The study design has been described more extensively previously [29]. We showed already the involvement of multiorgan failure during the clinical course of mechanically ventilated patients with SARS-CoV-2 infection in the current MaastrICCht cohort [30].

Briefly, The MaastrICCht cohort included all participants with respiratory insufficiency requiring mechanical ventilation and at least a PCR positive for SARS-CoV-2 and/or a chest computed tomography (CT) scan suggestive for SARS-CoV-2 infection, based on a CORADS-score of 4–5 [29, 31]. After training by qualified research staff and daily supervision by a senior investigator, medical research interns and PhD candidates not involved in patient care included participants. They collected clinical, physiological, and laboratory variables using a predefined study protocol (extensively described elsewhere) [29]. More specifically, the doses of thromboembolic prophylaxis and therapeutic low molecular weight heparin (LMWH) or unfractionated heparin (UFH) were collected each day. As evidence of high risk of thromboembolic complications in SARS-CoV-2 increased throughout the pandemic [6], the thromboembolic prophylaxis dose of nadroparin (i.e. LMWH used) for the MaastrICCht cohort was increased over time as follows: < 70 kg 2850 U, 70-90 kg 3800 U, > 90 kg 5700 U until April 1st; < 70 kg 3800 U, 70-90 kg 5700 U, > 90 kg 7600 U until April 23rd; and < 70 kg 5700 U, 70-90 kg 7600 U, > 90 kg 11,400 U after April 23rd in line with the consensus statement of the Dutch Association of Internal Medicine [32]. Patients with an indication for therapeutic anticoagulation received a fixed LMWH dose over the entire period of 3800 U, 5700 U or 7600 U twice a day according to their body weight and renal function. Patients on renal replacement therapy or mechanical circulatory support were treated with UFH. For other drugs such as deep muscle relaxants, the administration per day was categorised as yes or no, instead of the dose. For the present study, we investigated a sub-cohort of the MaastrICCht cohort, including patients undergoing diagnostic tests for suspected pulmonary embolism.

The local institutional review board (Medisch Ethische Toetsingscommissie (METC) 2020–1565/ 300,523) of the Maastricht UMC+ approved the study. The study is registered in the Netherlands Trial Register (registration number NL8613). Data for the present study were collected from March 25th until May 17th, 2020 (i.e., the full first wave of COVID-19 patients in our centre).

### Biomarker measurements

Venous blood was drawn daily between 4.30–5.30 a.m. and collected using 2.7 mL BD 3.2% citrate and 5.0 mL BD serum Vacutainer® vacuum tubes. Concentrations of fibrinogen and D-dimer were measured within 2 h of blood collection in citrated plasma, using a Sysmex CS2100i haemostasis analyser (Sysmex Corporation, Kobe, Hyogo, Japan). Detectable fibrinogen concentration had a maximum of 9 g/L. Concentrations of CRP (CRP, third generation, Roche Diagnostics, Basel, Switzerland) and ferritin (Elecsys ferritin, Roche) were measured on the COBAS®8000 by Roche Diagnostics in serum.

### Outcome variables

Patients were classified with or without a clinical pulmonary thromboembolism as follows; In patients in which clinical pulmonary thromboembolism was suspected, computed tomography pulmonary angiography (CTPA) was used to diagnose pulmonary thromboembolism. CTPA was performed in a supine position after intravenous injection of individually adapted contrast volume (iopromide 300 mg iodine; Ultravist, Bayer Healthcare, Berlin, Germany) based on body weight and kVp settings) on a second or third-generation dual-source CT scanner (Somatom Definition Flash, Force; Siemens Healthineers, Forchheim Germany). The protocol has been described in detail elsewhere [33, 34]. The image quality of all CT scans was judged sufficient for evaluation of the presence of pulmonary embolism or thrombosis (central, lobular, segmental or subsegmental). In addition, a right-sided cardiac thrombus diagnosed by transthoracic ultrasound (TTU) was scored as a clinical pulmonary thromboembolism [35]. Furthermore, when hemodynamic instability did not allow clinical CTPA, therapy with recombinant tissue plasminogen activator was scored as clinical pulmonary thromboembolism. Patients in whom CTPA excluded pulmonary thromboembolism were classified as not having clinical pulmonary thromboembolism. The occurrence of deep venous thrombosis (DVT) diagnosed by ultrasound was recorded within the cohort but has not been included as the primary outcome, as it was largely biased, as hospital infection prevention policy restricted the use of ultrasound during the first pandemic wave. The incidence of DVT was probably underestimated.

### Statistical analyses

The data were analysed with R version 3.6.1. The sample characteristics were described using mean and standard deviation (SD), median and interquartile range (IQR), or percentage, as appropriate.

First, the cohort participants were categorised into patients with clinical pulmonary thromboembolism (CTPA positive, TTU positive for cardiac thrombus and therapy with recombinant tissue plasminogen activator) and patients not having clinical pulmonary thromboembolism (CTPA negative). Next, we used linear mixed-effects regression with a random intercept and random slope for time to compute average differences in fibrinogen, D-dimer, CRP and ferritin over time and differences in the slope over time between both groups. When the difference in the slope over time between groups was not statistically significant, models for average differences are presented. Specifically, we used an unstructured variance-covariance matrix and an autoregressive correlation structure of the first order for longitudinal measures. To assess non-linear change over time, we added polynomials of time. Using the Akaike Information Criterion, the best fitting model for change over time was selected.

We computed unadjusted group differences in fibrinogen, D-dimer, CRP and ferritin (Model 1). Next, the model was adjusted for age, sex, APACHE-II score, BMI and a daily dose of LMWH or daily use of UFH (yes/no) (Model 2). Furthermore, models 2 for fibrinogen and D-dimer were additionally adjusted for CRP and ferritin, and models 2 for CRP and ferritin were additionally adjusted for fibrinogen and D-dimer. We also tested for effect-modification of the association between fibrinogen, D-dimer, CRP and ferritin over time and outcome by sex by adding a three-way interaction term to the models.

### Additional analyses

To investigate potential confounding by the presence of diabetes mellitus and other cardiovascular risk factors, a history of active use of therapeutic anticoagulants, and daily use of deep muscle relaxants during mechanical ventilation (yes/no), model 2 was additionally adjusted for these variables.

As a sensitivity analysis, we re-analysed the data, excluding patients with TTU positive for cardiac thrombus and therapy with recombinant tissue plasminogen activator, comparing CTPA positive with CTPA negative patients. Besides, we re-analysed models for D-dimer, CRP and ferritin after log-transformation because these variables were skewed. Finally, we compared baseline characteristics of all patients included in the main analyses with those from the full cohort who were excluded, as they did not undergo a CTPA during admission.

## Results

### Demographics

**Fig. 1:**
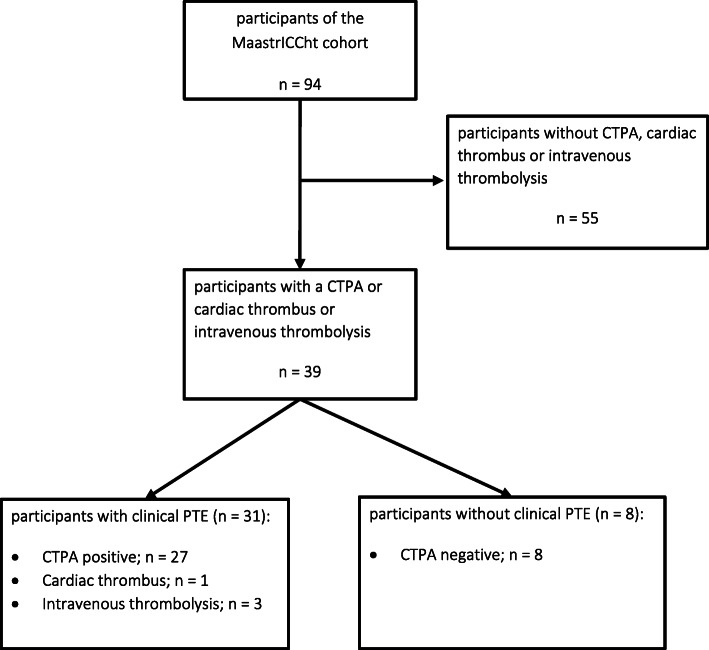
Flowchart patient population. Legenda: flow diagram of MaastrICCht cohort patients included in the present study. MaastrICCht: Maastricht Intensive Care COVID; CTPA = computed tomography pulmonary angiogram; PTE: pulmonary thromboembolism

Of a total of 94 *MaastrICCht* cohort participants, 27 had a CTPA confirmed clinical pulmonary thromboembolism, 1 had a right atrial thrombus on TTU, and 3 had therapy with recombinant tissue plasminogen activator. In 8 participants, pulmonary thromboembolism was ruled out by CTPA (Fig. 1). In the full cohort, 2 participants developed a DVT but were not suspected of pulmonary thromboembolism and excluded from the present analyses. Thus, the present study reports 39 participants with (*n* = 31) and without (*n* = 8) pulmonary thromboembolism. The characteristics of the included participants are presented stratified by primary outcome (Table 1). The mean age was 61.6 ± 12.9 years, 87.1% were men (mean age for men was 63.7 ± 10.9 years, and for women was 58.8 ± 20.6 years). APACHE-II score on admission was 15.7 ± 4.4 for participants with clinical pulmonary thromboembolism, compared to 19.0 ± 7.5 for those without clinical pulmonary thromboembolism (*p*-value 0.13).

**Table 1:** General characteristics

Variables	Pulmonary Thromboembolism			No Pulmonary Thromboembolism	
	Total (*n* = 31)	CT-confirmed (*n* = 27)	Suspected (*n* = 4)	CT-confirmed (*n* = 8)	***p***-value(Total vs No event)
Age, year, Mean (SD)	61.6 (12.9)	62.4 (13.4)	56.8 (9.0)	65.1 (11.7)	0.57
Male, (%)	27 (87.1)	23 (85.2)	4 (100.0)	6 (75.0)	0.58
Height, cm, Mean (SD)	177.2 (9.2)	176.0 (9.2)	185 (4.1)	171.0 (9.4)	0.10
Weight, kg, Mean (SD)	83.0 (11.0)	82.0 (10.4)	90.0 (14.1)	89.0 (19.1)	0.58
Body mass index, kg/m^2^, Mean (SD)	26.4 (2.8)	26.5 (2.8)	26.2 (3.7)	30.6 (6.9)	0.10
Admission location, by transfer from other hospital					0.41
Emergency room, n (%)	12 (38.7)	11 (40.7)	1 (25.0)	2 (25.0)	
Hospital ward, n (%)	11 (35.5)	9 (33.3)	2 (50.0)	5 (62.5)	
Transfer from another ICU, n (%)	8 (25.8)	7 (25.9)	1 (25.0)	1 (12.5)	
Prior anti-coagulant use, yes (%)	1 (3.2)	1 (3.7)	0 (0)	0 (0)	1
Diabetes and complications, yes (%)	2 (6.5)	2 (7.4)	0 (0)	1 (12.5)	0.51
Presence of cardiovascular risk factors (hypertension, dyslipidaemia, smoking, obesity), yes (%)	14 (45.2)	11 (40.7)	3 (75.0)	2 (25.0)	0.43
APACHE- II score, points, Mean (SD)	15.7 (4.4)	15.4 (4.2)	17.5 (5.3)	19.0 (7.5)	0.13

*SD* standard deviation; *ICU* Intensive Care Unit; *APACHE* Acute Physiology and Chronic Health Evaluation

**Fig. 2:**
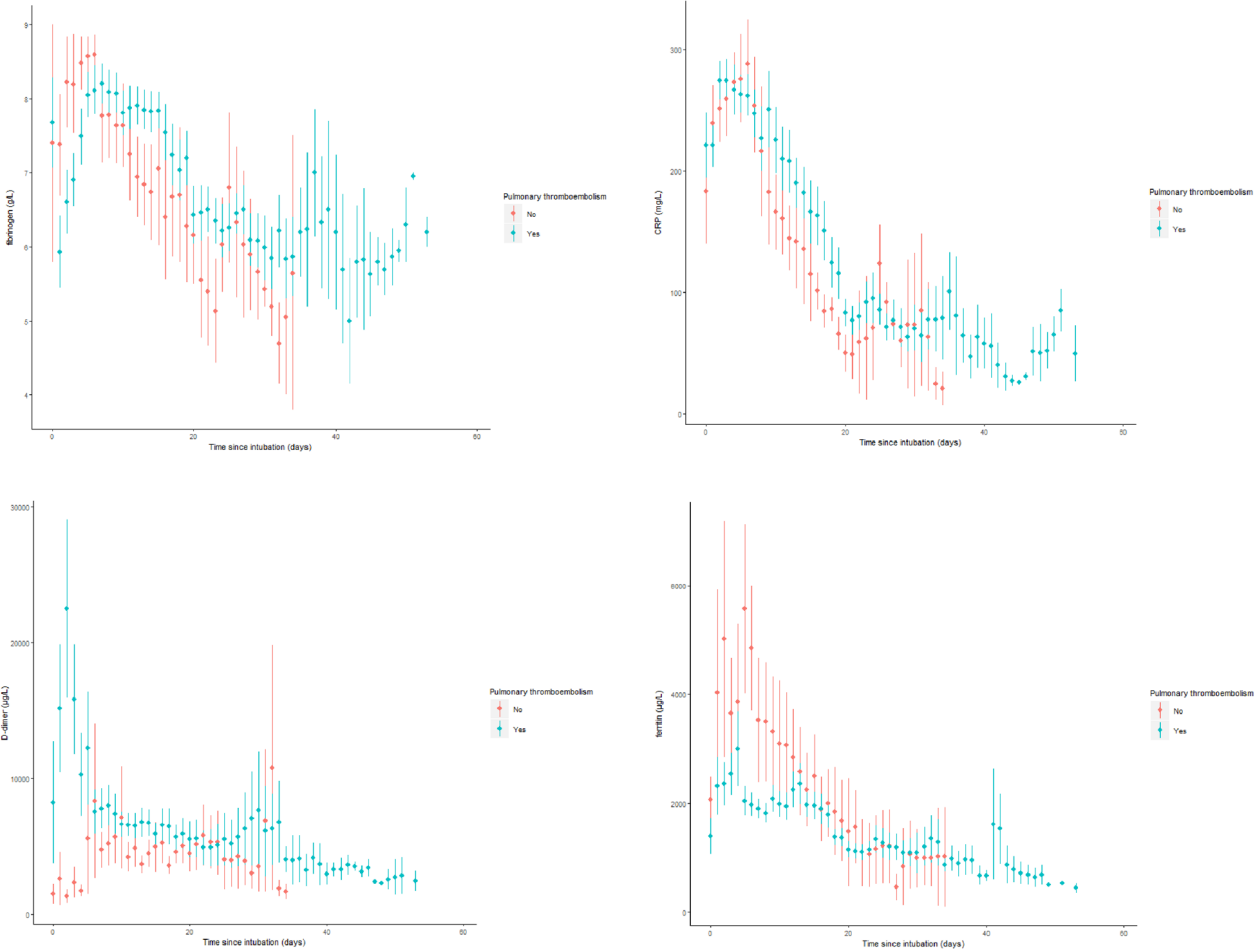
Evolution of crude concentrations (mean ± SD, on days since the date of intubation) of fibrinogen, D-dimer, CRP and ferritin over time for a patient suffering from clinical pulmonary thromboembolism or not

Unadjusted concentrations of fibrinogen, D-dimer, CRP and ferritin were initially all elevated but were decreased over time (Fig. 2).

### Associations between biomarkers and clinical pulmonary thromboembolism

Fibrinogen: In the crude model, fibrinogen was not associated with clinical pulmonary thromboembolism (Table 2, model 1). After adjustment for sex, age, APACHE-II score, BMI, and a daily dose of LMWH and UFH use, patients with clinical pulmonary thromboembolism, had, on average over time, a statistically significantly lower concentration of − 0.9 (95% CI: − 1.6; − 0.1) g/L (*p* = 0.030) compared to those without (Table 2, model 2). There was no statistically significant interaction with time since intubation observed on fibrinogen levels between the groups, and regression coefficients for average differences over time are reported only. Further adjustment of model 2 by CRP and ferritin did not change the result (Table 2, model 3).

**Table 2:** The linear mixed-effects models show the difference in average fibrinogen, D-dimer, CRP, and ferritin concentration development over time in mechanically ventilated patients with and without clinical pulmonary thromboembolism

Model	Regression coefficient (95% CI) Fibrinogen (g/L)	***p***-value	Regression coefficient (95% CI) D-Dimer (μg/L)	***p***-value	Regression coefficient (95% CI) CRP (mg/L)	***p***-value	Regression coefficient (95% CI) Ferritin (μg/L)	***p***-value
**Model 1: Crude**								
No clinical PTE (reference)	Ref.	Ref.	Ref.	Ref.	Ref.	Ref.	Ref.	Ref.
Presence of clinical PTE ^a^	0.2 (− 0.8–1.2)	0.672	2040 (− 4655–8735)	0.541	28 (− 25–80)	0.295	− 714 (− 1666–238)	0.138
**Model 2: Model 1 additionally adjusted for sex, age, Apache -II score, BMI (continuous, kg/m**^**2**^**) and nadroparin dosing (dose in units), and unfractionated heparin usage (yes/no)**								
No clinical PTE (reference)	Ref.	Ref.	Ref.	Ref.	Ref.	Ref.	Ref.	Ref.
Presence of clinical PTE ^a^	−0.9 (− 1.6 – − 0.1)	0.030	561 (−6212–7334)	0.868	27 (−32–86)	0.359	−1045 (− 1983 – − 106)	0.031
**Model 3 additionally adjusted for CRP and ferritin or fibrinogen and D-dimer**								
No clinical PTE (reference)	Ref.	Ref.	Ref.	Ref.	Ref.	Ref.	Ref.	Ref.
Presence of clinical PTE ^a^	−0.8 (−1.6–0)	0.061	3304(− 1969–8577)	0.214	19 (−37–75)	0.494	−592 (− 1426–242)	0.161

*PTE* pulmonary thromboembolism; *CI* confidence interval; *APACHE* Acute Physiology and Chronic Health Evaluation; *BMI* body mass index

^a^A negative regression coefficient indicates that the fibrinogen concentration is, on average, lower over time compared to the reference group

D-dimer: The development (both average and slope over time) of D-dimer concentration did not differ over time between patients with clinical pulmonary thromboembolism compared to those without (Table 2, models 1–3).

CRP: The development (both average and slope over time) of CRP concentration did not differ over time between patients with clinical thromboembolism compared to those without (Table 2, models 1–3).

Ferritin: In a crude model, ferritin was not associated with clinical pulmonary thromboembolism (Table 2, model 1). After adjustment for sex, age, APACHE-II score, BMI, and a daily dose of LMWH and UFH use, patients with clinical pulmonary thromboembolism compared to those without, had on average, a statistically significantly lower concentration of − 1045 μg/L (95% CI: − 1983 – − 106, *p* = 0.031) (Table 2, model 2). The slope (i.e., change in fibrinogen) over time between both groups did not differ. Further adjustment of model 2 by fibrinogen and D-dimer reduced the strength of the association, and statistical significance was lost (− 592 μg/L (95%CI: − 1426 – 242, *p* = 0.161) (Table 2, model 3).

### Effect modification by sex

We observed a statistically significant interaction between sex and fibrinogen, indicating that the association between fibrinogen and the occurrence of clinical pulmonary thromboembolism differed between men and women. Results stratified by sex showed that men with clinical pulmonary thromboembolism had a statistically significantly lower concentration of fibrinogen of − 1.3 (− 2.2; − 0.4) g/L (*p* = 0.006) compared to those without whereas no associations were present in women (Table 3, model 3). No statistically significant interaction of sex for the associations between D-dimer (p for interaction = 0.465), CRP (p for interaction = 0.875), or ferritin (p for interaction = 0.351) and clinical pulmonary thromboembolism were present.

**Table 3:** Stratified for men and women. The linear mixed-effects models show the average difference in fibrinogen, D-dimer, CRP, and ferritin concentration development over time in mechanically ventilated patients with and without clinical pulmonary thromboembolism

Model	Regression coefficient (95% CI) Men (*n* = 33) Fibrinogen (g/L)	***p***-value	Regression coefficient (95% CI) Women (*n* = 6) Fibrinogen (g/L)	***p***-value
**Model 1: Crude**				
No clinical PTE (reference)	Ref.	Ref.	Ref.	Ref.
Presence of clinical PTE *	−0.05 (− 1.2–1.1)	0.926	0.425 (−1.4–2.3)	0.564
**Model 2: Model 1 additionally adjusted for age, sex, Apache -II score, BMI (continuous, kg/m**^**2**^**) and nadroparin dosing (dose in units), and unfractionated heparin usage (yes/no)**				
No clinical PTE (reference)	Ref.	Ref.	Ref.	Ref.
Presence of clinical PTE ^a^	−1.3 (− 2.2 – − 0.4)	0.006	1.3 (− 11.6–14.2)	0.427
**Model 3: Model 2 additionally adjusted for CRP and ferritin or fibrinogen and D-dimer**				
No clinical PTE (reference)	Ref.	Ref.	Ref.	Ref.
Presence of clinical PTE ^a^	−1.2 (−2.2 – −0.3)	0.011	1.5 (− 10.4–13.5)	0.363

*PTE* pulmonary thromboembolism; *CI* confidence interval; *APACHE* Acute Physiology and Chronic Health Evaluation; *BMI* body mass index; *CRP* C-reactive-protein

^a^A negative regression coefficient indicates that the fibrinogen concentration is overall lower over time compared to the reference group

### Additional analyses

When we re-analysed the data excluding patients with TTU positive for cardiac thrombus and therapy with recombinant tissue plasminogen activator, comparing only CTPA positive (*n* = 27) with CTPA negative (*n* = 8) patients, the results did not materially change (data not shown). When we re-analysed the data and log-transformed D-dimer and CRP, the results did not materially change, whereas the association for ferritin was not statistically significant in any of the models (*p* = 0.133, for model 2, data not shown). Finally, the baseline characteristics of all patients included in the main analyses (*n* = 39) did not differ from those (*n* = 55) of the full cohort who were excluded as they did not undergo a CTPA during admission (Supplemental Table 1).

Additional analyses with step-by-step adjustments showed that the APACHE-II score, indicating disease severity at admission, was the main driver for change in direction for the association between fibrinogen and pulmonary embolism (Supplemental Table 2, models 1–3). When we additionally adjusted models 2 for the presence of diabetes mellitus and cardiovascular risk factor, and history of active use of therapeutic anticoagulants, and daily use of deep muscle relaxants during mechanical ventilation, results did not materially change (Supplemental Table 2, models 4–6).

## Discussion

In the present study, we analysed a sub-cohort of the MaastrICCht cohort, including 39 patients on mechanical ventilation with SARS-CoV-2 infection [36]. We classified two groups: one group with proven clinical pulmonary thromboembolism and one without clinical pulmonary thromboembolism. The study has four main findings. First, compared to patients without clinical pulmonary thromboembolism, those with clinical pulmonary thromboembolism had a lower fibrinogen concentration on average over time, after adjustment for sex, age, APACHE-II score, BMI, and a daily dose of LMWH and unfractionated heparin use. In particular, the adjustment for disease severity (APACHE-II score) appeared to affect the association between fibrinogen and pulmonary embolism. This association was unchanged after adjustment for inflammatory biomarkers CRP and ferritin. Second, the increase or decrease in fibrinogen concentration was not associated with clinical pulmonary thromboembolism. Third, the association for fibrinogen is apparent in men, whereas results in women are less pronounced possibly due to the small sample of women. Finally, no associations were observed for higher D-dimer, higher CRP or higher ferritin concentration and clinical pulmonary thromboembolism. However, we observed a lower ferritin concentration, in patients with, compared to those without, clinical pulmonary thromboembolism; the strength of this association was reduced and lost statistical significance after adjustment for fibrinogen and D-dimer. In addition, a sensitivity analysis using log-normalised ferritin concentrations was also not statistically significant.

The MaastrICCht cohort (*n* = 94) has an incidence of at least 33% for clinically relevant pulmonary thromboembolism in line with others (incidence 22–30%) who reported on SARS-CoV-2 infected patients admitted to the Intensive Care [37–40]. Based on the sub-cohort of patients who were selected for CTPA, the number is even more striking, as 27/35 ICU patients (77%) had pulmonary thromboembolism in this respect.

The occurrence of pulmonary thromboembolism and its association with coagulation and inflammatory biomarkers has been studied previously in SARS-CoV-2 [37, 39, 40]. Garcia-Olivé and colleagues reported serial D-dimer concentrations concerning the occurrence of pulmonary thromboembolism or not in SARS-CoV-2. They showed that non-ICU, SARS-CoV-2 positive patients with higher concentrations of D-dimer have an increased risk for pulmonary thromboembolism [38]. However, these results were not adjusted potential confounders (e.g., age, sex, APACHE-II score, BMI, a daily dose of LMWH and UFH use, and the presence of diabetes mellitus and cardiovascular risk factors, and history of active use of therapeutic anticoagulants).

Fibrinogen concentrations in our study population were elevated above the normal range (2–4 g/L) during their stay at the Intensive Care (Fig. 2 panel a). However, concentrations were, on average, over time, lower in patients with, compared to those without, clinical pulmonary thromboembolism. Next to its central role in clot formation, fibrinogen is also known as an acute-phase protein that is up-regulated during inflammation [41]. A high concentration of fibrinogen above the normal range has been associated with coagulopathy and endothelial damage, both present in SARS-CoV-2 [42–45]. On the other hand, next to its primary function in coagulation, fibrinogen interacts with platelets, endothelial cells and extracellular proteins, a process enhanced during acute inflammation [7, 46]. A possible explanation for these findings could be that patients with pulmonary thromboembolism may consume more fibrinogen in the process of thrombosis and/or pulmonary embolism. Tang and colleges reported a similar mechanism in non-ICU admitted SARS-CoV-2 infected patients [47]. The mechanism of fibrinogen consumption with clot formation has been proposed in non-SARS-CoV-2 patients [21, 24]. Moreover, autopsy studies in SARS-CoV-2 showed the presence of diffuse fibrinogen deposits in pulmonary vessels and microthrombi, suggesting local deposition or production of fibrin [44, 48–50]. Interestingly, the widespread formation of thrombi has been proposed as a physiological inflammatory host response to prevent the dispersion of harmful pathogens [51].

D-dimer concentrations above the normal range (> 500 μg/L) are associated with pulmonary thromboembolism and poor prognosis in SARS-CoV-2 [37–40, 52]. In the present study, increased D-dimer concentrations over the course of mechanical ventilation were not associated with clinical pulmonary thromboembolism. However, when looking at Fig. 2 panel B, we cannot exclude the possibility that differences early in the course of mechanical ventilation for SARS-COV-2 infection may point to increased thromboembolic risk.

Earlier studies reported that a higher D-dimer concentration was associated with a higher risk for pulmonary thromboembolism for patients presented at the emergency department or admitted to the general ward [53, 54]. However, adjustment for confounders was limited in these studies [53, 54]. Oudkerk et al. recommended using cut-off D-dimer values in consideration of applying CTPA in SARS-CoV-2 patients who are suspected of pulmonary thromboembolism [13]. However, the present results suggest that high concentrations of D-dimer, serially measured, do not discriminate between the presence or absence of clinical pulmonary thromboembolism in mechanically ventilated patients admitted to the ICU. Importantly, our crude and adjusted results for fibrinogen show that confounding plays a significant role, and this precludes the use of crude biomarker concentrations for clinical decision-making.

We observed high D-dimer concentrations in all patients. Moreover, D-dimer values were not able to discriminate between the presence of clinical pulmonary thromboembolism or not. The latter suggests the possible imbalance between coagulation and fibrinolytic turnover in SARS-CoV-2 infection. Indeed, suppression of fibrinolysis has been described during acute lung injury, including acute respiratory distress syndrome (ARDS) and SARS-CoV-1 [55–58]. Whether therapeutic targeting of the fibrinolytic system in SARS-CoV-2 infection is advantageous remains to be investigated [59–61]. The sub-cohort of the MaastrICCht cohort, including patients undergoing diagnostic tests for suspected pulmonary embolism, a-priori has a high risk of pulmonary embolism (i.e. 77%), as compared to the previous populations studied [1–5]. In fact, these patients had higher average concentrations of biomarkers over time compared to the patients of the MaastrICCht cohort not suspected of pulmonary embolism (Supplemental Table 1). This could partly explain our observation that D-dimer concentrations over time were not associated with pulmonary embolism.

In SARS-CoV-2 infection, the coagulation system is likely activated and dysregulated due to an acute inflammatory response [42, 45]. Ferritin has been described as a contributing factor in the cytokine storm syndrome presumed to play a role in severe SARS-CoV-2 infection [26]. However, the present data show that both CRP and ferritin were not higher in patients with as compared to those without a clinical pulmonary thromboembolism.

In contrast, Al-Samkari et al. showed significantly increased ferritin and CRP levels in patients who developed a thrombotic complication [10]. They included patients for possible venous thromboembolism (deep venous thrombosis and pulmonary embolism) based on radiological confirmation and predefined clinical criteria. However, they did not rule out the presence of venous thromboembolism in their control group, which we did in the present study. Furthermore, they included patients with arterial thrombosis and non-vessel thrombotic complications in their study group as well. Only a minority (*n* = 144 ICU admitted versus *n* = 256 non-ICU admitted) of the included participants by Al-Samkari were critically ill, suggesting a considerable diversity of disease severity. As noted previously, disease severity is an important aspect in the reflection of biomarkers (e.g., ferritin and CRP are elevated in ICU admitted patients diagnosed with SARS-CoV-2 compared to patients admitted to the regular ward) [62]. In the present study, only critically ill patients admitted to ICU were included. This may explain why our results for CRP and ferritin were not associated with clinical pulmonary thromboembolism and differ from other reports, such as the one by Al-Samkari et al [10].

Differences between men and women characterise thrombosis and haemostasis physiology; for example, sex hormones regulate procoagulant specific gene expression and altered platelet and vascular function in women [63]. In addition, the reproductive state in women plays a crucial role [64]. However, except during pregnancy, normal D-dimer and fibrinogen levels do not differ between men and women [65, 66]. Our colleagues of the MaastrichCCht consortium and the COVID-Data-Platform (CoDaP) already proposed the added value of considering sex differences in SARS-CoV-2 [67]. However, we found a significantly lower concentration of fibrinogen in men, as compared to women, with clinical pulmonary thromboembolism. However, this result should be interpreted with caution, as the number of women in the group with clinical pulmonary thromboembolism is relatively low.

The major strengths of the present study are the serial measurements of biomarkers of coagulation and inflammation and the CTPA diagnosis and exclusion of clinical pulmonary thromboembolism within a well-defined prospective cohort study [36]. The extensive characterisation allowed us to adjust for potentially confounding variables while using state-of-the-art multi-level data analysis techniques for serial data. The multi-level data analysis techniques have the advantage of including all available data from intubation to discharge independently of patient transfers between Intensive Care hospitals due to logistical reasons caused by the pandemic.

The study has some limitations. First, during the SARS-CoV-2 pandemic, we did not systematically screen for the occurrence of DVT as a hospital infection prevention policy restricted the use of ultrasound during the first pandemic wave. The incidence of DVT in the MaastrICCht cohort is 2% and thereby likely underestimated. Several other studies reported a low incidence of DVT [6, 68, 69]. In these studies, the use of venous ultrasound might also have been restricted due to logistical reasons during a pandemic crisis. The incidence of DVT might, therefore, be underestimated in SARS-CoV-2 [70–77]. The exact pathobiology of thrombotic complications in SARS-CoV-2 is largely unclear, although microvascular thrombo-inflammation appears to play a role [44, 78–81]. The detection of diffuse microthrombi in pulmonary microvasculature appears challenging using regular CTPA. Alternative methods such as subtraction CT angiography appear more promising in SARS-CoV-2 [82]. Moreover, radiological parameters obtained from CTPA as right ventricular to left ventricular ratio were already proposed in SARS-CoV-2 [83]. High-quality CTPA using fast data acquisition and dedicated reconstruction parameters are advantageous in this respect. Taken together, based on the above, we speculate that the classical form of venous thromboembolism does not fully explain the occurrence of thrombotic complications in SARS-CoV-2. Consequently, a specific therapeutic approach for SARS-CoV-2 related thrombo-inflammation might even be required. Second, not each patient within the full cohort underwent a CTPA, which is inherent to the design of the clinical observational study. Furthermore, the indication for CTPA was based on clinical decision-making. To further minimise the chance of selection bias in the reported associations, we performed a sensitivity analysis with CTPA confirmed and excluded patients only, which showed similar results. Moreover, the overall characteristics of the participants in the current analysis did not differ from the other patients of MaastrICCht cohort, except for a higher ferritin concentration (Supplemental Table 1).

## Conclusion

Mechanically ventilated patients infected with SARS-CoV-2 have a profound thrombo-inflammatory biomarker profile over time. Fibrinogen was, on average, significantly lower in patients with pulmonary thromboembolism compared to patients without clinical pulmonary thromboembolism. D-dimer, CRP and ferritin concentrations were not associated with clinical pulmonary thromboembolism. This contributes to evidence suggesting that endothelial fibrin deposition and possibly impaired fibrinolytic functions play a role in SARS-CoV-2 and the presence of clinical pulmonary thromboembolism. A more comprehensive analysis of the coagulation system, for example, using rotational thromboelastometry (ROTEM/tPA-ROTEM) and thrombin generation (TG), might be required to unravel thrombo-inflammation in SARS-CoV-2 induced coagulopathy further.

## Supplementary Information

**Additional file 1: Supplemental Table 1**. General characteristics of the Maastricht Intensive Care COVID (MaastrICCht) cohort. **Supplemental Table 2**. The linear mixed-effects models, with step-by-step adjustments, show the difference in fibrinogen, d-dimer, c-reactive protein, and ferritin concentration development over time between mechanically ventilated patients developing a confirmed or suspected pulmonary thrombotic event in comparison with patients lacking this development. Patients who had been discharged to the ICU of another hospital were omitted.

## Data Availability

The datasets during and/or analysed during the current study available from the corresponding author on reasonable request.

## Abbreviations

APACHE-II: Acute physiology and chronic health evaluation II
ARDS: Acute respiratory distress syndrome
BMI: Body mass index
CI: Confidence interval
CORADS: COVID-19 reporting and data system
CoDap: Consortium and the COVID-data-platform
CRP: C-reactive-protein
CT: Computed tomography
CTPA: CT pulmonary angiography
DVT: Deep venous thrombosis
ICU: Intensive care unit
IQR: Interquartile range
LMWH: Low molecular weight heparin
MaastrICCht: Maastricht intensive care cohort
Maastricht UMC: Maastricht university medical centre
METc: Medisch ethische toetsingscomissie
PCR: Polymerase chain reaction
PTE: Pulmonary thromboembolism
ROTEM: Rotational thromboelastometry
tpa-ROTEM: Tissue plasminogen activator-ROTEM
SARS-CoV-2: Severe acute respiratory syndrome coronavirus 2
SD: Standard deviation
TG: Thrombin generation
TTE: Transthoracic echocardiography
UFH: Unfractionated heparin
