# Supplementary material for: Serial markers of coagulation and inflammation and the occurrence of clinical pulmonary thromboembolism in mechanically ventilated patients with SARS-CoV-2 infection; the prospective Maastricht intensive care COVID cohort
Source: Thromb J. 2021 May 31;19:35. doi: 10.1186/s12959-021-00286-7 (PMC8165953; doi:10.1186/s12959-021-00286-7)
Supplement: Supplementary file 1 — Additional file 1: Supplemental Table 1. General characteristics of the Maastricht Intensive Care COVID (MaastrICCht) cohort. Supplemental Table 2. The linear mixed-effects models, with step-by-step adjustments, show the difference in fibrinogen, d-dimer, c-reactive protein, and ferritin concentration development over time between mechanically ventilated patients developing a confirmed or suspected pulmonary thrombotic event in comparison with patients lacking this development. Patients who had been discharged to the ICU of another hospital were omitted. [file 12959_2021_286_MOESM1_ESM.docx]

Supplemental Table 1. General characteristics of the Maastricht Intensive Care COVID (MaastrICCht) cohort.

| **Variables** | **Sub-cohort group** (n = 39) | **Excluded group**  (n = 55)* | **p-value** |
| --- | --- | --- | --- |
| Age, year, Mean (SD) | 62.4 (12.6) | 65.6 (11.3) | 0.20 |
| Male, (%) | 33 (84.6) | 39 (73.6) | 0.31 |
| Height, cm, Mean (SD) | 175.9 (9.4) | 174.6 (8.6) | 0.51 |
| Weight, kg, Mean (SD) | 84.2 (13.0) | 85.2 (13.9) | 0.73 |
| Body mass index, kg/m^2^, Mean (SD) | 27.3 (4.2) | 27.9 (4.1) | 0.47 |
| Admission location, by transfer from other hospital |  |  | 0.75 |
| Emergency room, n (%) | 14 (35.9) | 17 (32.1) |  |
| Hospital ward, n (%) | 16 (41.0) | 20 (37.7) |  |
| Transfer from another ICU, n (%) | 9 (23.1) | 16 (30.2) |  |
| Anticoagulant use, yes (%) | 1 (2.6) | 3 (5.7) | 0.64*** |
| Diabetes and complications, yes (%) | 3 (7.7) | 11 (20.8) | 0.15 |
| Presence of cardiovascular risk factors (hypertension, dyslipidaemia, smoking, obesity), yes (%) | 16 (41.0) | 26 (49.1) | 0.58 |
| APACHE-II score, points, Mean (SD) | 16.4 (5.3) | 15.3 (6.0) | 0.36 |
| Fibrinogen, g/L, median (IQR) | 7.0 (2.2) | 7.2 (2.0) | 0.98 |
| D-dimer, µg/L, median (IQR) | 6350 (6923) | 4692 (6363) | 0.10**** |
| CRP, mg/L, median (IQR) | 170.5 (111.4) | 154.9 (97.8) | 0.52**** |
| Ferritin, µg/L, median (IQR) | 1644 (1482) | 1200 (995) | 0.04**** |
| ICU Mortality, n (%)** | 16 (43) | 18 (41) | 0.83*** |
| Length of ICU stay, days, median (IQR)** | 16 (14) | 18.5 (19.5) | <0.001 **** |

SD: standard deviation; ICU: Intensive Care Unit; APACHE: Acute Physiology and Chronic Health Evaluation; CRP: C-reactive-protein.

* missing data (n=2).

** until discharge, transfer or death.

*** Fisher's exact test instead of Chi-square test.

**** Mann-Whitney U test.

Supplemental Table 2. The linear mixed-effects models, with step-by-step adjustments, show the difference in fibrinogen, d-dimer, c-reactive protein, and ferritin concentration development over time between mechanically ventilated patients developing a confirmed or suspected pulmonary thrombotic event in comparison with patients lacking this development. Patients who had been discharged to the ICU of another hospital were omitted.

| **Model** | **Regression**  **coefficient (95% CI)** | **p-value** |  | | | **Regression**  **coefficient (95% CI)** | **p-value** | | |  | | **Regression coefficient (95% CI)** | | **p-value** |  | **Regression coefficient (95% CI)** | **p-value** | | |  |  |
| --- | --- | --- | --- | --- | --- | --- | --- | --- | --- | --- | --- | --- | --- | --- | --- | --- | --- | --- | --- | --- | --- |
|  | **Fibrinogen (g/L)** |  |  | | | **D-Dimer (µg/L)** | |  |  | | **CRP (mg/L)** | |  | |  | **Ferritin (µg/L)** | |  |  |  |  |
| **Model 1: Crude** | | | | | | | | | | | | | | | | | | | |  |  |
| No clinical PTE (reference) | Ref. | Ref. | |  | Ref. | | Ref. | | |  | | Ref. | | Ref. |  | Ref. | Ref. | | |  |  |
| Presence of clinical PTE * | 0.20 (-0.75 – 1.16) | 0.672 | |  | 2040  (-4655 – 8735) | | 0.541 | | |  | | 28 (-25 – 80) | | 0.295 |  | -714 (-1666 – 238) | 0.138 | | | | |
| **Model 2: Model 1 additionally adjusted for age and sex** | | | | | | | | | | | | | | | | | | | | |  |
| No clinical PTE (reference) | Ref. | Ref. | |  | Ref. | | Ref. | | |  | | Ref. | | Ref. |  | Ref. | Ref. | | | | |
| Presence of clinical PTE * | 0.17 (-0.80 – 1.14) | 0.724 | |  | 2164 (-4504 – 8831) | | 0.515 | | |  | | 32 (-20 – 83) | | 0.218 |  | -852 (-1754 – 50) | 0.064 | | | | |
| **Model 2.1: Model 2 additionally adjusted for APACHE-II score** | | | | | | | | | | | | | | | | | | | | |  |
| No clinical PTE (reference) | Ref. | Ref. | |  | Ref. | | Ref. | | |  | | Ref. | | Ref. |  | Ref. | Ref. | | | | |
| Presence of clinical PTE * | -0.81  (-1.50 – -0.12) | 0.023 | |  | 3195  (-3327 – 9716) | | 0.327 | | |  | | 30  (-23 – 83) | | 0.264 |  | -874  (-1706 – -43) | 0.041 | | | | |
| **Model 3: Model 2 additionally adjusted for APACHE-II score, BMI (continuous, kg/m^2^) and nadroparin dosing (dose in units) and unfractionated heparin usage (yes/no)** | | | | | | | | | | | | | | | | | | | | |  |
| No clinical PTE (reference) | Ref. | Ref. | |  | Ref. | | Ref. | | |  | | Ref. | | Ref. |  | Ref. | Ref. | | | | |
| Presence of clinical PTE * | -0.85 (-1.62 – -0.10) | 0.030 | |  | 561 (-6212 – 7334) | | 0.868 | | |  | | 27 (-32 – 86) | | 0.359 |  | -1045 (-1983 – -106) | 0.031 | | | | |
| **Model 4: Model 3 additionally adjusted for BMI, diabetes mellitus and presence of cardiovascular risk factors** | | | | | | | | | | | | | | | | | | | | |  |
| No clinical PTE (reference) | Ref. | Ref. | |  | Ref. | | Ref. | | |  | | Ref. | | Ref. |  | Ref. | Ref. | | | | |
| Presence of clinical PTE * | -0.94 (-1.74 – -0.148) | 0.023 | |  | 661 (-6234 – 7556) | | 0.847 | | |  | | 27 (-33 – 87) | | 0.369 |  | -965 (-1906 – -23) | 0.047 | | | | |
| **Model 5: Model 3 additionally adjusted for history of active use of therapeutic anticoagulants** | | | | | | | | | | | | | | | | | | | | |  |
| No clinical PTE (reference) | Ref. | Ref. | |  | Ref. | | Ref. | | |  | | Ref. | | Ref. |  | Ref. | Ref. | | | | |
| Presence of clinical PTE * | -1.1 (-1.9 – -0.3) | 0.007 | |  | 666 (-6182 –7515) | | 0.845 | | |  | | 23 (-35 – 81) | | 0.426 |  | -1041 (-1996 – -86) | 0.035 | | | | |
| **Model 6: Model 3 additionally adjusted for daily use of deep muscle relaxants** | | | | | | | | | | | | | | | | | | | | |  |
| No clinical PTE (reference) | Ref. | Ref. | |  | Ref. | | Ref. | | |  | | Ref. | | Ref. |  | Ref. | Ref. | | | | |
| Presence of clinical PTE * | -0.87 (-1.63 – -0.11) | 0.027 | |  | 694 (-6033 – 7422) | | 0.836 | | |  | | 29 (-30 – 88) | | 0.330 |  | -1058 (-1986 – -129) | 0.028 | | | | |
|  |  |  | |  |  | |  | | |  | |  | |  |  |  |  | | | | |

PTE: pulmonary thromboembolism; CI: confidence interval; APACHE: Acute Physiology and Chronic Health Evaluation; BMI: body mass index.

^*^A negative regression coefficient indicates that the fibrinogen concentration is, on average, lower over time compared to the reference group.
